# Evaluation of a Community-Based Falls Prevention Program in South Florida, 2008-2009

**Published:** 2012-12-15

**Authors:** Anamica Batra, Michael Melchior, Laura Seff, Newman Frederick, Richard C. Palmer

**Affiliations:** Robert Stempel College of Public Health and Social Work, Florida International University; Florida International University, Miami, Florida; Florida International University, Miami, Florida; Florida International University, Miami, Florida; Florida International University, Miami, Florida

## Abstract

**Introduction:**

Many older adults experience fear of falling, which may reduce participation in routine activities. A Matter of Balance (MOB) and Un Asunto de Equilibrio (ADE) workshops were offered in South Florida to reduce fear of falling and increase activity levels in older adults. The objectives of this study were to evaluate the effectiveness of the lay leader model of the programs in the first year of their implementation and to further report on participant outcome measures.

**Methods:**

We analyzed reach, adoption, and implementation data for participants who attended workshops between October 1, 2008, and December 31, 2009, who were aged 60 years or older, and who had both baseline and posttest outcome data. Workshops were in English and Spanish and consisted of 8 two-hour sessions. Participants completed a 7-item baseline and posttest questionnaire that consisted of a falls management scale, a social activity item, and modified version of Physician-Based Assessment and Counseling on Exercise. We analyzed outcome data on multiple characteristics using a general linear model. A class evaluation questionnaire measured participant satisfaction.

**Results:**

Results for 562 participants who provided both baseline and posttest data showed significant improvement on 6 of 7 questions for MOB and all questions for ADE (*P* < .001). The 391 participants who provided evaluation data indicated that the programs were effective, beneficial, and well organized.

**Conclusion:**

Lay leaders successfully implemented the programs in community settings. The programs were effective in reducing fear of falling among older adults.

## Introduction

Unintentional falls are a significant predictor of illness and death in older adults (1-4). Evidence suggests that 1 in 3 older adults in the United States falls at least once each year (1-4). According to the Centers for Disease Control and Prevention (CDC), approximately 30% of the total number of people with a recorded fall in 2009 were aged 60 years or older (5). Among older adults, unintentional falls are the most common cause of all nonfatal injuries (1,2,4,6-8).

In addition to actual falls, many people experience a fear of falling. Lach (9) found an adjusted prevalence of 30.6 per 100 for fear of falling in older adults, which increased to 47.2 over 2 years. People who have a fear of falling are twice as likely to fall compared to those without. They also experience more anxiety and social isolation (10). Furthermore, fear of falling can affect quality of life (11,12) through restricted activities and increased frailty, leading to poorer health outcomes (13).

The primary goal of A Matter of Balance (MOB) and its Spanish equivalent, Un Asunto de Equilibrio (ADE), is to empower people who have fallen or who fear falling with a sense of control over falls. Expected outcomes include increased activity levels and improved mobility. The program uses cognitive strategies to address the factors associated with falls in older adults (14). Although the success of MOB has been documented (15,16), no evidence has been published for ADE. Additionally, results that correspond with the adoption and practical application of these programs by community-based agencies are lacking. Therefore, the primary objective of the study was to evaluate the success and effectiveness of the lay leader model of both MOB and ADE in community-based settings in South Florida. A secondary objective was to further report on participant outcome measures for MOB and to report ADE outcome measures for the first time.

## Methods

### Study design and setting

We used a pretest-posttest study design to evaluate outcomes of MOB and ADE. Various community-based agencies offered MOB and ADE through grants from the Health Foundation of South Florida (HFSF) as part of the Healthy Aging Initiative. The goal of the Healthy Aging Initiative, created by HFSF, is to increase local community capacity to make health promotion programs accessible to older adults in South Florida.

**Box.** Overview of A Matter of Balance (MOB) and Un Asunto de Equilibrio (ADE) Falls Prevention Programs, South Florida, 2008-2009

**Measures**	MOB	ADE
No. of participants	160	402
No. (%) of completers	150 (93.8)	388 (96.5)
No. of workshops offered	35	48
No. of sites	9	27
No. of instructors^a^	14	29
No. (%) of workshops with recommended attendance of ≥8 h	12 (34.3)	33 (68.8)

a Some program instructors taught in both the programs.

In this study, an older adult is defined as a person aged 60 or older. According to US Census 2000 data, older adults make up 22.2% of South Florida's population (17). As part of the Healthy Aging Iniative, HFSF funded 5 agencies serving older adults in Broward, Miami-Dade, and Monroe counties of South Florida to offer MOB and ADE to older adults between October 1, 2008, and December 31, 2009. Agencies delivered 83 workshops (35 MOB and 48 ADE) at 36 sites (Box). Most agencies chose adult day care, older adult centers, clinics, hospitals, skilled nursing facilities, assisted living facilities, community centers, or housing for older adults as sites for program delivery.

### MOB/ADE program overview

MOB and ADE workshops consisted of 8 two-hour sessions offered once or twice per week for a total of 16 hours of class time. A master-trainer or trained lay leaders led the sessions. Recommended class size was 8 to 14 participants. Initial sessions focused on helping participants learn to view falls and fear of falling as controllable. Later, the instructors introduced simple exercises aimed at improving strength and balance. They helped participants set realistic goals for changing attitudes and discussed ways to modify their environment to reduce falls and the fear of falling. Finally, participants were encouraged to continue exercise after finishing the workshop.

### Instructor training

Each provider agency and its partner agencies identified master-trainers and several lay leaders for training. External methods used to recruit trainers included word of mouth, flyers, and advertisements. In some cases, MOB and ADE program participants decided to become lay leaders or master-trainers after completing a workshop.

Master-trainer training required 2 days of training with comprehensive curriculum material. The training focused on skills needed to recruit and train MOB lay leaders, coordinate and market the programs in the community, and evaluate outcomes. Lay leader training, conducted by the Partnership for Healthy Aging (PFHA) or PFHA-trained master-trainers, included review of information about older adults and the specific program emphasis. Training focused on learning the program script and the detailed class outline, key elements of fidelity assurance. Lay leaders also got supervised teaching experiences with live workshops.

The Healthy Aging Initiative trained an initial group of master-trainers and lay leaders within the first 3 months of program implementation. Local master-trainers conducted 3 additional trainings to increase the cadre of lay leaders. Thirty master-trainers and 54 lay leaders were trained.

### Participant recruitment

Qualified participants were people aged 60 or older who were able to solve problems and were interested in improving flexibility, balance, and strength. Agencies that offered the workshops recruited participants by targeting their existing client base and other areas with a high density of residents older than 60. The program was promoted with informational flyers, posters, and word of mouth.

### Data collection

We measured reach, adoption, and implementation. Reach was defined as the number of participants and completers in each program. A completer was defined as a participant who attended at least 5 of 8 sessions, including the last session (16). Adoption measures included number of workshops offered, number of sites and instructors used, and number of workshops that met minimum recommended attendance as a percentage of all workshops offered. Implementation addressed fidelity (ie, consistency in delivery of the program as intended). To assess fidelity, we observed 30% of each agency's workshops. Assessment of program effectiveness focused on the effect of the program on targeted outcome measures.

On the first day of the workshop, participants completed 1) a consent form, 2) a participant information form that recorded demographic information, and 3) a baseline questionnaire to assess outcome measures. The baseline questionnaire consisted of 7 items and included the Falls Management Scale (FMS) and items that assessed social activity level and readiness to exercise. The FMS, developed in 1998 by Tennstedt et al, consists of 5 items and measures a person's self-perceived ability to manage actual falls or risk of falls (15,16). When used in randomized controlled trials, the scale has a reported Cronbach alpha reliability of 0.76 to 0.84, and for the lay leader model its reported Cronbach alpha is 0.85 to 0.87 (15,16). Responses used a 4-point Likert scale (1 = not at all sure to 4 = very sure). A higher score indicated more confidence in handling falls and less fear (15,16).

Consistent with previous falls prevention studies, a 5-point Likert scale item assessed whether fear of falling interfered with social activity (1 = not at all to 5 = interfered extremely) (15). The original Physician-Based Assessment and Counseling on Exercise (PACE) tool evaluates the readiness of a person to exercise (18) in the context of 3 stages described in the transtheoretical model (19). Agencies used a modified version of the 11-item PACE tool, developed by CDC and used in a previous falls prevention study (15), to evaluate participants' readiness to exercise. The modified version includes the first 6 response categories from the original instrument and also assesses 3 stages of behavior change: precontemplation, contemplation, and preparation (15).

At the end of the 8th session, participants completed a posttest questionnaire, which included the same 7 items asked on the baseline questionnaire, and an evaluation questionnaire that assessed satisfaction with the program. All questionnaires were self-administered, although instructors and agency staff were generally available to help participants complete the questionnaires if necessary. Data collection instruments used to collect data from ADE participants were in Spanish.

Each agency received training and was responsible for entering all collected data into an online database within 2 weeks of completing a workshop. To ensure the data were reliable, we compared data entered in the online database with the original forms completed by participants for 30% of all workshops offered by each agency.

### Data analysis

Data were extracted from the online database for the period October 1, 2008, through December 31, 2009, imported into SPSS version 17.0 (SPSS, Inc, Chicago, Illinois), cleaned, and if necessary, reverse coded. First, we examined demographic characteristics of study participants. Next, we calculated the number of workshops offered, number of workshops that met minimum recommended attendance, and number of sites and instructors used. Then, we used a general linear model to test for differences between baseline and posttest across outcome measures, while controlling for potential site effects. We used a general linear model rather than multiple *t* tests to assess the difference in test scores to account for the common within-subject variance across items. Moreover, the use of multiple *t* tests to assess differences would increase type 1 errors. The 5 FMS items were analyzed separately. The PACE and social activity items were each analyzed as a single item measure. Participants who were younger than 60, who did not report their age at the time of data collection, or who had missing outcome data were not included in analysis.

## Results

A total of 958 older adults participated in either an MOB (n = 275) or ADE (n = 683) workshop. Based on inclusion criteria, 396 participants were excluded, leaving a sample of 562; (MOB, n = 160; ADE, n = 402). Of these, 538 were completers (Box). We did not find any significant demographic differences between excluded and included participants.

More women than men participated in both MOB and ADE (Table 1). Most MOB participants were non-Hispanic white, and most ADA participants were Hispanic. Few MOB participants but more than half of ADE participants reported an annual household income less than $15,000. Neither program attracted many participants who saw themselves as frail or disabled.

For both MOB and ADE, the FMS score increased significantly for each item at posttest (Tables 2 and 3). A significant increase in the posttest mean of the PACE measure in both study groups suggests that after attending the MOB and ADE workshops, participants' readiness to include exercise in their daily routine increased. We also found significant changes in mean for the social activity measure for participants in both groups.

One hundred and fifteen MOB participants and 276 ADE participants completed the evaluation questionnaire, a 72% and 69% response rate, respectively. All respondents either strongly agreed or agreed that the programs were effective, beneficial, and well organized. Most respondents (MOB, 87%; ADE, 89%) agreed that the instructors were well prepared for the class and the participant workbook helped them better understand the classes. Most participants (MOB, 84%; ADE, 86%) said that they would recommend the program to a friend or relative.

## Discussion

Overall, our findings suggest that community agencies were able to implement MOB and ADE and achieve desired participant outcomes. The number of MOB participants reached in our study is higher than in another falls prevention randomized controlled trial (16) but lower than a recent community-based study of MOB that used a volunteer lay leader model (15). No published study has reported evaluation findings on ADE.

Each agency that implemented MOB or ADE met their proposed goals of participant reach and number of workshops to be offered. In addition to increasing capacity for continuation of the MOB and ADE programs, the master and lay leader trainings represent a successful collaborative effort to share training responsibilities and make maximum use of available resources. The significant changes in program outcomes and the success of implementation as indicated by participant evaluations and fidelity observations, while not under research controls, provide further support for the effectiveness of the lay leader model of MOB. These study findings are similar to those reported elsewhere (15).

Participants significantly changed their attitudes about falling after completing the program. The significant change between baseline and posttest FMS scores indicates that the content and design of the programs helped participants increase their level of self-confidence in their ability to control falls and manage fear of falling. Other studies that assessed these outcomes in controlled settings and in community-based settings found similar results (15,16). Furthermore, change in a person's attitude toward sense of control over falls can lead to improvement in everyday function and engagement in low-impact physical activity, enhancing quality of life. Our interpretation is supported by other studies (20,21) that found that older adults who reported low control over falls also reported greater declines in ability to perform activities of daily living.

The PACE measured participants' comprehension of the importance of exercise and affirmation of readiness to perform and include exercises in their daily routines. Similar to other measures, PACE results also showed significant improvement for participants in both programs. Healy et al reported similar findings (15). Other studies document additional benefits of this attitude change toward exercise. These studies suggest that involvement of older adults in some kind of exercise helps to reduce rates of illness and death for many chronic conditions such as hypertension, stroke, diabetes, and obesity (20-22).

Overall, results showed changes from baseline to posttest in social activity scores for both MOB and ADE participants, although the change in mean of 0.12 for MOB participants is smaller than the 0.26 change documented in another community-based falls prevention program (15). The social activity item measures the degree to which participants believed that fear of falling interfered with their daily activity and ultimately reduced social activity, both of which have a well-established relationship with fear of falling (23,24). One study documented increase in anxiety and depression as the negative consequences of social isolation for older people (25). Therefore, the improvement on this item for both MOB and ADE suggests that this initiative was successful.

Our outcomes are consistent with those of previous MOB studies (15,16,26). Moreover, the positive and significant results in all ADE measures, and the large number of ADE participants, indicate that the Spanish version of the program was well received and that outcomes were consistent with those published for MOB.

This study has several limitations. First, it has limited generalizability because findings are for programs offered in South Florida. The agencies that offered the programs, and program participants, may not be representative of agencies and participants in other communities. Second, because the evaluation design had no control group, participant results may reflect other treatments or historical events. Third, participants were not randomly selected but rather volunteered because they thought they might benefit from the program. Therefore, selection bias may have skewed results. Similarly, all data collected were self-reported and therefore subject to response and social desirability biases. Finally, because of funding limitations and the fact that MOB had previously been deemed effective in reducing falls, data regarding actual falls before and after program participation were not collected (14-16,26,27). Future studies that look at the effect of covariates and moderator variables on outcome data and predictive factors are needed.

In summary, successful implementation of MOB and ADE through the Healthy Aging Initiative demonstrated that local capacity could be established to offer falls prevention programs to older adults in community settings. The model used by the Healthy Aging Initiative can be replicated by other communities that identify a need for falls prevention programs for older adults to improve their quality of life and to reduce the burden on the health care system. Our results support the premise that, when trained properly, lay leaders can implement evidence-based programs effectively, which opens the possibility of implementing such programs on a wider scale and with less cost in many locations in the United States.

## Figures and Tables

**Table 1:** Baseline Characteristics of Participants in A Matter of Balance (MOB) and Un Asunto de Equilibrio (ADE) Falls Prevention Programs, South Florida, 2008-2009^a^

**Characteristic**	MOB, n (%) (n = 160)	ADE, n (%) (n = 402)
**Sex**		
Female	130 (81.3)	351 (87.3)
Male	29 (18.1)	48 (11.9)
**Age, y**		
60-69	47 (29.4)	54 (13.4)
70-79	46 (28.8)	177 (44.0)
80-89	54 (33.8)	148 (36.8)
≥90	13 (8.1)	23 (5.7)
**County of residence**		
Broward	13 (8.1)	1 (0.2)
Miami-Dade	65 (40.6)	393 (97.8)
Monroe	81 (50.6)	0
**Race/ethnicity**		
Hispanic/Latino	50 (31.3)	383 (95.3)
Haitian/other non-Hispanic Caribbean	2 (1.3)	0
Non-Hispanic white	96 (60.0)	4 (1.0)
American Indian/Alaska Native	3 (1.9)	0
Asian	3 (1.9)	1 (0.2)
Other	0	1 (0.2)
**Primary language**		
English	116 (72.5)	9 (2.2)
Spanish	44 (27.5)	389 (96.8)
Other	0	4 (1.0)
**Marital status**		
Married/partnered	53 (33.1)	90 (22.4)
Single/not partnered	97 (60.6)	308 (76.6)
**Household size**		
Living alone/1-person household	90 (56.3)	274 (68.2)
Living with ≥1 other person	70 (43.8)	128 (31.8)
**Annual household income, $**		
<15,000	28 (17.5)	234 (58.2)
15,000-24,999	31 (19.4)	25 (6.2)
25,000-49,999	32 (20.0)	3 (0.7)
50,000-75,000	6 (3.8)	0
>75,000	2 (1.3)	0
**Education**		
Less than high school	17 (10.6)	173 (43.0)
High school	42 (26.3)	97 (24.1)
Some college	45 (28.1)	50 (12.4)
College graduate	52 (32.5)	66 (16.4)
**Other characteristics**		
Frail/disabled	12 (7.5)	32 (8.0)
Has Medicare	140 (87.5)	333 (82.8)
Has Medicaid	20 (12.5)	253 (62.9)

a Percentages do not total 100 because of missing data.

**Table 2:** Baseline to Posttest Differences for Study Participants (n = 160) in A Matter of Balance Falls Prevention Program, South Florida, 2008-2009^a^

**Characteristic**	Baseline, Mean (SD)	6-Week Posttest, Mean (SD)	Change in Mean^b^
**Falls management scale^c^**			
How confident are you that you can find a way to get up if you fall?	2.62 (0.98)	3.13 (0.85)	0.51
How sure are you that you can find a way to reduce falls?	2.59 (0.86)	3.19 (0.73)	0.60
How sure are you that you can protect yourself if you fall?	2.15 (0.79)	2.78 (0.84)	0.63
How sure are you that you can increase your physical strength?	2.80 (0.89)	3.25 (0.77)	0.45
How sure are you that you can become more steady on your feet?	2.68 (0.83)	3.22 (0.77)	0.54
**Social activity item^d^**			
During the last 4 weeks, to what extent has your concern about falling interfered with your normal social activities with family, friends, neighbors, or groups?	2.05 (1.02)	1.93 (1.14)	0.12
**PACE item^e^**			
How much are you walking or exercising now?	4.62 (1.56)	5.24 (1.09)	0.62

Abbreviations: SD, standard deviation; PACE, Physician-Based Assessment and Counseling on Exercise.

a Only questions with baseline and posttest responses were analyzed. Not all participants responded to all questions.

b All changes were significant at *P* < .001 (calculated by using the general linear model), except for the social activity item.

c Response scale: 1 = not at all sure; 4 = very sure.

d Original questionnaire had a response scale of 1 = not at all to 5 = extremely. This scale was reverse coded for analysis so that 5 = not at all and 1 = extremely.

e Item is modified PACE tool to assess readiness to exercise (18). Response scale: 1 = not at all/don't intend to; 2 = not regularly but may start; 3 = trying to start; 4 = exercised infrequently for more than 1 month; 5 = doing moderate exercise <3 times/wk; 6 = doing moderate exercise ≥3 times/wk.

**Table 3:** Baseline to Posttest Differences for Study Participants (n = 402) in Un Asunto de Equilibrio Falls Prevention Program, South Florida, 2008-2009^a^

**Characteristic**	Baseline, Mean (SD)	6-Week Posttest, Mean (SD)	Change in Mean^b^
**Falls management scale^c^**			
How confident are you that you can find a way to get up if you fall?	2.15 (0.99)	3.06 (0.85)	0.91
How sure are you that you can find a way to reduce falls?	2.27 (0.93)	3.18 (0.76)	0.91
How sure are you that you can protect yourself if you fall?	2.07 (0.94)	3.03 (0.83)	0.96
How sure are you that you can increase your physical strength?	2.29 (0.87)	3.17 (0.73)	0.88
How sure are you that you can become more steady on your feet?	2.36 (0.94)	3.20 (0.75)	0.84
**Social activity item^d^**			
During the last 4 weeks, to what extent has your concern about falling interfered with your normal social activities with family, friends, neighbors, or groups?	2.46 (1.15)	1.84 (1.01)	0.62
**PACE item^e^**			
How much are you walking or exercising now?	3.88 (1.63)	4.96 (1.30)	1.08

Abbreviations: SD, standard deviation; PACE, Physician-Based Assessment and Counseling on Exercise.

a Only questions with baseline and posttest responses were analyzed. Not all participants responded to all questions.

b All changes were significant at *P* < .001 (calculated by using general linear model).

c Response scale: 1 = not at all sure; 4 = very sure.

d Original questionnaire had a response scale of 1 = not at all to 5 = extremely. This scale was reverse coded for analysis so that 5 = not at all and 1 = extremely.

e Item is modified PACE tool to assess readiness to exercise (18). Response scale: 1 = not at all/don't intend to; 2 = not regularly but may start; 3 = trying to start; 4 = exercised infrequently for more than 1 month; 5 = doing moderate exercise <3 times/wk; 6 = doing moderate exercise ≥3 times/wk.
